# Student learning performance prediction based on online behavior: an empirical study during the COVID-19 pandemic

**DOI:** 10.7717/peerj-cs.1699

**Published:** 2023-11-17

**Authors:** Yiyi Liu, Zijie Huang, Gong Wang

**Affiliations:** 1 Department of Information, Mechanical and Electrical Engineering, Shanghai Normal University, Shanghai, China; 2 Department of Computer Science and Engineering, East China University of Science and Technology, Shanghai, China

**Keywords:** Educational data mining, Machine learning, Students’ performance, Online learning

## Abstract

In the context of the COVID-19 global pandemic, highly intense and frequent online teaching has leapt to be one of the dominant learning patterns and become an ordinary situation in university teaching practices. In recent years, progress in feature engineering and machine learning has made it possible for more effective educational data mining, which in turn has enhanced the performance of intelligent learning models. However, the potential impact of increasing and varying features on online instruction in this new situation makes it unclear whether the existing related findings and results are practical for teachers. In this article, we use various state-of-the-art machine learning techniques to predict students’ performance. Based on the validation of the rationality of the built models, the importance of features under different feature selection techniques are calculated separately for the datasets of two groups and compared with the features before and at the beginning of the pandemic. The results show that in the current new state of highly intense online learning, without considering student information such as demographic information, campus attributes (administrative class and teaching class) and learning behavior (completion of online learning tasks and stage tests) these dynamic features are more likely to discriminate students’ academic performances, which deserves more attention than demographics for teachers in the guidance of students’ learning. In addition, it is suggested that further improvements and refinements should be made to the existing features, such as classifying features more precisely and expanding in these feature categories, and taking into account the statistics about students’ in-class performances as well as their subjective understanding of what they have learned. Our findings are in line with the new situation under the pandemic and provide more implications to teachers’ teaching guidance.

## Introduction

In recent years, the COVID-19 pandemic has caused major disruptions in people’s work and life and posed a huge impact on a wide range of fields (Harper et al., 2020; Pokhrel & Chhetri, 2021; Dev & Sengupta, 2020). In the field of education, the spread of COVID-19 has resulted in traditional classroom teaching forced to shift to online teaching and learning and has become the new situation in college teaching practices. At the same time, the feasibility of online learning systems has made online teaching and learning method to be more widely accepted and more rapidly promoted, in which the field of educational data mining has become more active (Hernández-Blanco et al., 2019).

Educational data mining (EDM) is a significant subdomain in data mining that focuses on using data mining, machine learning, and statistical methods to solve problems in prior studyies and to facilitate discovery in educational settings (Baker & Yacef, 2009). Shabihi & Kim (2021) identified six topics and 19 subtopics for EDM. Predictive analytics which included academic success prediction, behavior prediction, and retention prediction is among them. As an effective tool, EDM technique is used in educational data to predict academic performance, analyze learning performance, and improve the learning/teaching environment (Yağcı, 2022). In these prediction tasks, student achievement and failure rate are often considered as one of the most reflective performance of student learning outcomes in colleges and universities. Therefore, predicting the learning process and analyzing students’ performance is considered a major task in the field of EDM (Tsiakmaki et al., 2019). Researchers often conduct studies from different perspectives such as students’ performance prediction (Buenaño-Fernandez, Villegas-CH & Luján-Mora, 2019), retention or dropout prediction (Buenaño-Fernandez, Villegas-CH & Luján-Mora, 2019; Musso, Hernández & Cascallar, 2020), learning progress (Asif et al., 2017; Baker, 2010), early prediction (Leite et al., 2021; Waheed et al., 2020) and influencing factors (Hussain, Khan & Ullah, 2022; Thiele et al., 2016; Li et al., 2020). Educational data such as student demographic information (gender, age, economic status, district, *etc*.) (Bernacki, Chavez & Uesbeck, 2020; Rizvi, Rienties & Khoja, 2019), educational records, historical grades, and classroom participation (Song & Li, 2021; Bonafini et al., 2017; Tomasevic, Gvozdenovic & Vranes, 2020) are often considered to predict students’ performance at the end of the school year. The continuous progress in feature engineering and machine learning has made it possible for more effective educational data mining, and models’ ability to predict online learning performance is improved. However, in the pandemic and post-pandemic era, intense online learning has led to a surge in online learning data, especially student learning behavior data, which has attracted the attention of teachers and education scholars. In this new situation, the need for online learning instruction for students in university has seen a soar in demand. It also can be well prepared for pandemic in the future. Whether there are undulation in the potential impact of different learning behaviors and attributes on students’ performance at the end of the semester has become a new issue, which also makes teachers wonder whether the original concerns and findings can be reused to instruct students.

**Figure 1:**
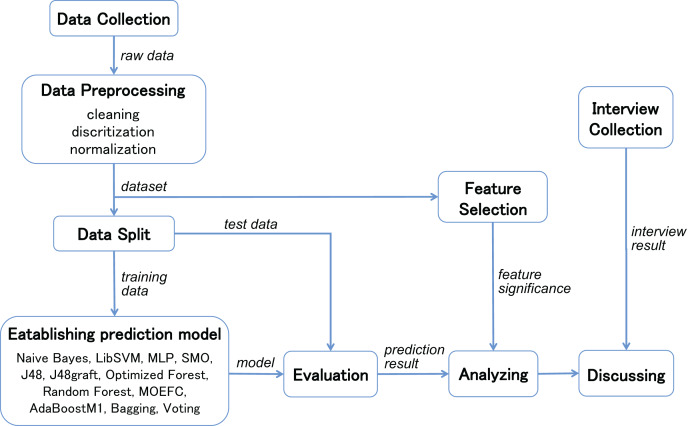
Procedure of establishing students’ performance prediction model and interpretation.

To address the issue mentioned above, we explore the features’ importance that may influence students’ final performance based in the context of the COVID-19 pandemic. In our study, we first select two similar language courses after filtering the Course Library, and implement classification for each of these two online teaching courses. The experiment evaluate the effectiveness of the selected techniques using several common metrics on the two different and independent courses data. Considering the current widely investigated EDM techniques, various state-of-the-art machine learning techniques and extended methods are considered in the study to predict students’ performance. Then, we use several algorithms in feature selection with different search method to rank the student online learning features. After combining interviews with teachers and students about their agreement with these features on online learning performance, we finally identified new important features that are instructive in online learning in the context of the COVID-19 pandemic. The procedure of the steps above is shown in Fig. 1.

The main contributions of this article are as follows:

1. We conduct a study on student learning behaviors for highly intense online learning during the pandemic.
2. We collect datasets from online courses in the COVID-19 pandemic, evaluate the performance of each technique, and achieve good performance.
3. Without considering demographic information, significant features which affect final academic performances have changed during the COVID-19 pandemic, which indicates that students’ learning behaviors may also change and deserve teachers’ attention.

The other parts of this article are as follows. “Related Work” summarizes the study related; “Dataset Collection and Preprocessing” introduces the construction of the dataset we used; “Methodology” describes relevant methods and technologies for this study; “Experiment” answers each RQ we propose; “Discussion” discusses the results of the experiment; “Threats to Validity” overviews the threats to the validity and our effort to cope with them; “Conclusion” concludes the article and describes future work.

## Related work

In EDM, some studies focus on the overall assessments of students such as the final grade, assessment results, and final marks. Among them, a few studies have also focused on the tasks of early prediction and clustering of students’ typical progress (Asif et al., 2017). Finding reliable and valid features that contribute significantly to performance is another challenge (Latif et al., 2021), in addition to finding generic prediction algorithms with higher accuracy and validity.

### Prediction models and performance

Xiao, Ji & Hu (2022) found that almost all (77 studies) of the 80 selected studies used supervised machine learning classification algorithms to predict students’ performance, and some (19) used ensemble method. More than one classifier was used in 69% of the selected models. Among them, the machine learning (ML) classifiers with more than 10 times are decision tree (DT), naive Bayes (NB), multi-layer perception (MLP), random forest (RF), support vector machine (SVM), logic regression (LR) and K-nearest neighbor (KNN), while the most used ensemble methods are classical boosting, bagging and voting. Shafiq et al. (2022) reviewed 100 papers on student prediction from 2017–2021. They also found that nearly 50% used supervised machine learning and RF, normal decision tree, LR, and NB was the top four algorithms. This was followed by deep learning at 28% (artificial neural network (ANN) and MLP). For our study, we need consider these prediction models to validate our dataset. This is because the difference of datasets often leads to different accuracy of the prediction models (Dutt, Ismail & Herawan, 2017; Rimpy, Dhankhar & Solanki, 2022). In light of the researches above, these commonly used algorithms will also appear in our experiments to predict students’ final grades for two similar undergraduate English courses.

### Features used in research

The features considered by models are often different in prior studies and some researchers have classified these features. Yağcı (2022) summarized the reviewed articles in which at least 14 features were found to be used. Abu Saa, Al-Emran & Shaalan (2019) identified nine types of factors influencing students’ performance by reviewing 36 studies, among which the most commonly used four types are students’ previous grades and class performance, students’ e-learning activity, students’ demographics, and students’ social information. Francis & Babu (2019) also divided the features affecting students’ performance into four categories, namely demographic features, academic features, behavioral features, and extra features.

Among these different kinds of features, demographic information are often included in the majority of studies. Fernandes et al. (2019) verified the ability of the relevant dataset variables to discriminate students’ performance by comparing the performance of the two datasets in the gradient boosting machine (GBM) with the receiver operating characteristic (ROC) curve of above 0.9. They found that neighborhood, school and age were potential indicators among the 17 variables that accounted for most of the demographics. Similarly, Cruz-Jesus et al. (2020) used 16 demographic data to predict the academic performance of students by machine learning techniques and RF, SVM, LR and kNN can achieve accuracies of 50–81%. Song & Li (2021) proposed an sequential engagement-based academic performance prediction network (SEPN) on the Open University Learning Analytics Dataset (OULAD) (Kuzilek, Hlosta & Zdrahal, 2017) containing demographic information. They validated the superiority of the SEPN by comparing it with seven existing methods. Following the SEPN, the SEPN-D (SEPN without involving demographic information) is the second best model.

However, there are also researches which prove that demographics are not important in predicting students’ performance. Tomasevic, Gvozdenovic & Vranes (2020) also selected the OULAD dataset to predict student test scores by different combinations of data types using classification and regression models. The results showed that demographic information did not significantly affect the accuracy of the predictions, while ANN obtained the highest overall accuracy with an F1-measure of 0.9662 and an RMSE of 12.1256. Yağcı (2022) obtain a good prediction performance without using demographic information. They proposed a model merely with midterm exam grades, faculty and department based on machine learning algorithms to predict the final exam grades of undergraduate students. Among the classifiers, NN and SVM have the highest classification accuracy. However, the final grade includes 40% of the midterm score in this study.

The major differences of our work to prior studies are: we involve two dynamic categories of online learning behavior and campus attributes to build predictors, rather than student demographic information. This is because the former dynamic features can provide more entry points for teachers to adjust the timely instruction and improve course quality, while the latter has very little guidance for classroom teaching during the pandemic and post-pandemic.

Furthermore, as the most used dynamic feature, viewing situation of videos (quantity and time) and learner characteristics often have a positive impact on students in the course and continuous learning (Butt et al., 2023). Videos can elicit positive perceptions of the students to promote the learning efficiency of the students. Students also benefit from the videos in terms of their understanding of course content and their participation in class discussion (Shek et al., 2023).

### Feature importance affecting student learning performance

For the predicted results, the quality of the selected features can be more important than the quantity (Ortiz-Lozano et al., 2020). Although there is a plethora of studies given students’ use of autonomous learning strategies towards scholastic achievement during the COVID-19 lockdown internationally, studies in the Asia region are still rudimentary and student interactive engagement and study environment have a significant impact on students’ scholastic achievement during the lockdown (Anthonysamy & Singh, 2023).

Roslan & Chen (2022) concluded from a review of 58 studies that the factors influencing students’ performance, as the focus of nearly half of the studies, included student academic record (accounting for 34% of all compiled aspects), student demographics (26%), course attributes (11%), with the remaining facets examined (*i.e*., student activities, students behavior, student psychological aspects). Shafiq et al. (2022) counted the number of occurrences of different features in 100 reviewed studies, giving the insignificant features, including attendance, gender, D.O.B/age, family size, parents’ education, and time spent in LMS log data. It is also noted that demographic information, as a static variable, has been shown to be less statistically significant in forecasting models. In our study, we try to find which factor can reflect student online-learning performance in those dynamic features. We use feature selection to solve this problem and then interview these students and teachers about their agreement with features and extra explanation for the features they chose.

As is mentioned above, this study bases on the context of the COVID-19 pandemic selects different algorithms such as commonly used machine learning, deep learning, and integrated learning to predict students’ final grades for two similar undergraduate English courses, which contain a large amount of online student learning data on learning behaviors. After validating the model, different feature selection algorithms are chosen to test the degree of influence of the collected features on the final prediction results in order to identify potentially significant features.

## Dataset collection and preprocessing

This section determines which features of the data are used and whether the data collected are appropriate for the purpose. Data preprocessing involves data scaling and reducing the redundant features that could be not valid to predict particular outcomes.

### Dataset collection

The study is conducted in two similar language courses in the required general education courses of a university during the COVID-19 pandemic in China, for all undergraduate students in a given grade. Teaching videos and courseware (PowerPoint slides, *etc*.) are made by teachers in advance and transmitted to the video platform in the corresponding courses arranged in the syllabus. Students can take the course online. When students study online, their video viewing, homework completion, and chapter quizzes are automatically recorded by the system. The final course result is determined by the teacher according to the students’ academic performance and examination results.

We collect student online learning data during the early stage of the pandemic as the prime dataset (Course A and Course B). When the students adapt to the studying mode, the construct of the courses are not changed, and we include the online learning data from this period as an extended dataset (Course A Extended and B Extended).

In the records of the prime dataset, 228 students taking the course A 129 students taking the course B are selected as two groups. In the record of extended dataset, 554 students taking the Course A Extended and 508 students taking the Course B Extended are selected as two groups. The end-of semester achievement scores, campus attributes and online learning behavior of the two groups of students are taken as datasets corresponding to the two courses. The variables in the raw datasets are shown in Table 1.

**Table 1:** Variables in raw datasets of two courses.

Type	Variables	Discription
Student info	Login_id	Student ID
	Stu_name	Student name
Campus attributes	Department_id	Department ID
	Department_name	Department name
	Class_id	Administrative class ID
	Class_name	Administrative class name
	Clazz_id	Teaching class ID
	Clazz_name	Teaching class name
Online learning behaviors	Job_num	Number of mission points completed (*e.g*., ppt of a section of the course)
	Job_rate	Rate of mission points completed
	Videojob_num	Number of video watched
	Videojob_time	Total length of video watched (minutes)
	Videojob_rate	Rate of video watched
	Test_num	Number of chapter quiz or homework completed
	Test_rate	Rate of chapter quiz or homework completed
	Test_avgscore	Average chapter quiz score
	Exam_num	Number of exams completed
	Exam_rate	Rate of exams completed
	Exam_avgscore	Average exam score
	pv	Number of visits to the online learning platform
	Sign_num	Number of sign-in completed
	Sign_rate	Rate of sign-in completed
	Special_time	Course topics reading hours
	…	…
Online learning performance	Final score	Final exam score

The end-of semester achievement score is a combination of final exam scores and class performance scores, ranging from 0 to 100. There are approximately 17 weeks (4.5 months) during the entire curriculum. In other words, the answer to the question how effective the online learning performance throughout the semester is on students’ performance at the end of the semester is investigated.

### Preprocessing

The experiment is validated by a classification task. Students’ final performance in the school is the final exam scores, which need to be divided in advance in the classification task. After referring to the rules for score-to-GPA conversion in university and other literature, we set an interval for each tenth of the score based on the pass line of 60, namely [90–100], [80–90], [70–80], [60–70] and [0–60].

In this part, first the features related to the students’ personal information (login_id and stu_name) are removed. For the features expressing the same meaning (department_name, class_name and clazz_name), the existing corresponding features with id are used instead. Subsequently, for each course, we discretize the name variables and normalized the continuous variables. Given the current number of features, no further filtering in these selected valid features. This is the same as the majority of studies reviewed in Xiao, Ji & Hu (2022), which shows 45 out of 80 studies used all features.

After data preprocessing, the identified features used finally in courses A and B are shown in Table 2. Finally, we split the prepared dataset into a training set and a test set in the ratio of 8:2.

**Table 2:** The identified features used finally in two courses.

Course	Type	Features
Course A (extended)	Campus attributes	Department_id, major_id, class_id, clazz_id
	Online learning behaviors	Videojob_time, pv, special_time, job_num, videojob_num
Course B (extended)	Campus attributes	Department_id, major_id, class_id, clazz_id,
	Online learning behaviors	Videojob_time, pv, special_time, job_num, videojob_num, test_num, test_avgscore, exam_num, exam_avgscore, sign_num

## Methodology

This section introduces the educational data mining techniques and related feature selection techniques involved. We use the WEKA tool to apply the EDM techniques analyzed in this experiment.

### Machine learning techniques

For comparison, we reproduce a number of existing algorithms and models to predict students’ final exam performance: naive Bayes, LibSVM, multi-layer perception (MLP), SMO (sequential minimal optimization algorithm for training a support vector classifier), J48, J48graft, optimized forest, random forest, multi objective evolutionary fuzzy classifier (MOEFC), AdaBoostM1, Bagging and Voting. To characterize the effectiveness of the EDM technique analyzed in this experiment, we decide to adopt accuracy in the classification task. In addition, in order to ensure the stability of evaluation and quantitative indicators, we average the indicators calculated by several independent tests of each analysis technique and obtain the results.

### Feature selection

Feature selection techniques we use consist of four filter-based feature ranking techniques and one filter-based feature subset selection techniques. Filter based feature selection techniques uses statistics measure the importance of each feature towards the class labels, rather than predetermine classification models and performance indicators. The overview of these six techniques are demonstrated in Table 3.

**Table 3:** The overview of six feature selection techniques.

Family	Methods		Abbreviation
Filter-based feature ranking	Statistics-based techniques	Correlation	CORR
	Probability-based techniques	Information gain	InfoGain
		Gain ratio	GainRat
	Instance-based techniques	ReliefF	ReF
Filter-based feature subset selection	Correlation-based feature subset selection	Best first	CorrBF
		GreedyStepwise	CorrGS

#### Filter-based feature ranking

Filter-based feature ranking techniques sort features by calculating the important score for each feature. Features with stronger correlation with class labels have higher scores. Statistic-based, probability-based and instance-based techniques are applied in the study.

- **Correlation (CORR) **(Guyon & Elisseeff, 2003) evaluates the worth of each features by calculating the correlation (Pearson’s) between it and the class.
- **Information gain (InfoGain) **(Cover, 1999) uses class labels to evaluate the reduction of uncertainty when given a specific feature. The disadvantage of InfoGain is that a multi-valued feature tends to obtain a higher InfoGain value.
- **GainRatio (GainRat) **(Quinlan, 2014) evaluates the worth of each features by calculating the gain ratio with respect to the class which takes punishment to the feature with more values against the disadvantage of InfoGain.
- **ReliefF(ReF**) (Kononenko, 1994) evaluates the worth of an features by repeatedly sampling an instance and considering the value of the given attribute for the nearest instance of the same and different class.

#### Filter-based feature subset selection

Instead of evaluating each feature separately, the filter-based feature subset selection technique evaluates the feature subset selected from the original feature set.

**Correlation-based feature subset selection (Corr) **(Hall, 2000) uses the correlation measure to select subsets of features that are highly correlated with the class while having low intercorrelation are preferred.

**Best first (BF) and greedy stepwise (GS)** are two kinds of search strategies to generate the feature subset with the correlation-based methods, and are employed in our study. As a heuristic search algorithm, BF obtains a feature subset with the hillclimbing and backtracking greedily. As a greedy search algorithm, GS generates a feature subset forward or backward and stops until the performance deterioration.

Combining the correlation-based feature subset selection technique and the two search strategies above, we have the following two techniques, including Corr with BF and Corr with GS, short for CorrBF and CorrGS individually.

## Experiment

In this section, we propose three research questions and answer them by demonstrating and discussing the results of our experiment. Written informed consent was obtained from all the participants prior to the enrollment (or for the publication) of this study.

### Experimental design

The new datasets mentioned above are different from other datasets in terms of the category of features included, *i.e*., it has a greater emphasis on being relevant to student learning and being adjustable. Thus, the goal of our study is to evaluate whether the students’ online learning data has an influence on student’s learning performance, for the purpose of providing teachers with suggestions on improving the quality of classroom teaching to implement in the COVID-19 pandemic. To these ends, we propose three research questions:

- **RQ 1**: Can the model we choose effectively predict with online learning data?
- **RQ 2**: Which features contribute the most predictive power, and what are the differences between them and those in other studies?
- **RQ 3**: Are the model behavior perceived as reasonable by course teachers in practice?

For RQ 1, we choose machine learning algorithms, which have been introduced in previous section. Considering the bottleneck problem of single model could be improved by the ensemble learning model, we also use well-established algorithms such as AdaBoost, bagging, and select some other algorithms in Weka. Meanwhile, the model parameters are adjusted according to their predicted performance to achieve the best performance. We compared our datasets with those in other literature listed in Table 4, and selected the intersection. The experimental results of the RF model on this intersection, namely campus attributes in our dataset, are chosen as baseline. After the experiment on the prime dataset, the prediction on the extended dataset of the same model is used to further verify the experiment.

**Table 4:** Features category of the related work in student online learning environment.

Dataset	Feature type	Feature name	Feature description
OULAD, Song & Li (2021), Tomasevic, Gvozdenovic & Vranes (2020)	Demographic	Gender, living environment, age, highest education	Students’ information
	Learning behavior	Sum_click	The number of times a student interacts with the material in that day
		Date	The date of student’s interaction with the material measured as the number of days
		TMA	Tutor marked assessment
		CMA	Computer marked assessment
Student-drop-india	Demographic	Gender, caste, guardian	Students’ information
	Campus attributes	Internet	Whether the student studies online
		School_id	The school which the student is in
		Science_teacher	The teacher of science
		Languages_teacher	The teacher teaching languages
	Learning behavior	Mathematics_marks	The score of mathematics
		English_marks	The score of english
		Science_marks	The score of science
	Other	Total_students	The number of students in the school
		Total_toilets	The number of toilets in the school
		Establishment_year	Year of establishment of the school
Students’ academic performance dataset (xAPI-EduData)	Demographic	Gender, nationality, birth place, parent responsible	Students’ information
	Campus attributes	Educational stages	Educational level student belongs
		Grade levels	Grade student belongs
		Section ID	Classroom student belongs
		Topic	Course topic
		Semester	School year semester
	Learning behavior	Raised hand	How many times the student raises his/her hand on classroom
		Visited resources	How many times the student visits a course content
		Viewing announcements	How many times the student checks the new announcements
		Discussion groups	How many times the student participate on discussion groups
		Student absence days	The number of absence days for each student
	Other	Parent answering survey	Parent answered the surveys provided from school or not
		Parent school satisfaction	The degree of parent satisfaction from school
Yağcı (2022)	Campus attributes	Department	The department the student belong
		Faculty	The faculty the student belong
	Learning behavior	Midterm exam grades	The midterm exam grade of the student
Fernandes et al. (2019)	Demographic	Gender, age, benefit, city, neighborhood	Students’ information
	Campus attributes	School	The school the student belong
		Subjects	The subject of the school
	Learning behavior	Absence	The times of absence for the student
		Grade	Grades for the first two months
	Other	Regional coordination education, School administrative region, Student with special needs, Class with special needs,Shift, Classroom usage environment	Other information in this study
Costa et al. (2017)	Demographic	Age, gender, civil status, city, income, student registration	Students’ information
	Campus attributes	Period, campus, class, semester, year of enrolling	Information in the campus
	Learning behavior	Performance	performance in the weekly activities and exams
		Frequency	access frequency of the student in the system
		Forum	participation in the discussions forum
		Files	amount of received and viewed files
		Quiz	complement of the quiz
	Other	Message, wiki, glossary, use of educational tools status on discipline	Other information in this study
Cruz-Jesus et al. (2020)	Demographic	Portuguese citizenship, Portuguese naturality, Gender, Age, Rural residence area, Population density, Economic level	Students’ information
	Campus attributes	Year of the study cycle	Information in the campus
		Number of enrolled years	
		Number of failures	
		Financial support level	
		Class size (students)	
		School size (students)	
	Learning behavior	Unit course	Number of unit courses attended in the presen year
	Other	Availability of a PC at home, internet access, scholarship	Other information in this study

For RQ 2, feature selection is not further implemented inpreprocessing, but is expanded as an experiment to evaluate the effect of different features on the prediction results. In addition, the results of different feature selection algorithms are comprehensively considered to obtain the importance of these features.

For RQ3, we examine whether the important features mentioned in RQ2 are practically meaningful through interviews. Our interviewees include students and instructors of both courses, and since the number of instructors is small compared to students, instructors of other similar language courses also serve as interviewees to provide their perceptions. The features used in each of the two courses in the experiment appear in the interviews through corresponding descriptions, which are intended to facilitate a better understanding by the interviewees, and allow the interviewee to select half of these features that he or she considers important for final performance. These evaluations are further analized to validate the results in RQ2, which could help teachers give students guidance in studying.

### Experimental results

#### RQ1: model performance

Tables 5 to 8 list the predicted performance and baseline of our selected models in both courses and course extended.

**Table 5:** Predictive performance of the selected model in course A.

	ACC	Precision	Recall	F-measure	MCC	AUC
Baseline (subset with RF)	67.391%	0.603	0.674	0.635	0.174	0.673
Naive Bayes	71.698%	0.651	0.717	0.663	0.222	0.744
LibSVM	78.261%	0.833	0.783	0.729	0.428	0.615
MLP	71.739%	0.732	0.717	0.704	0.285	0.721
SMO	78.261%	0.833	0.783	0.729	0.428	0.617
J48	70.588%	0.636	0.706	0.662	0.242	0.614
J48graft	71.739%	0.711	0.717	0.684	0.398	0.647
Optimized forest	70.627%	0.626	0.717	0.660	0.273	0.711
RF	71.739%	0.622	0.717	0.662	0.313	0.691
MOEFC	78.261%	0.834	0.783	0.736	0.477	0.643
AdaBoostM1	73.913%	0.648	0.739	0.659	0.330	0.693
Bagging	78.261%	0.833	0.783	0.729	0.428	0.615
Voting	78.261%	0.833	0.783	0.729	0.428	0.581

**Table 6:** Predictive performance of the selected model in course B.

	ACC	Precision	Recall	F-measure	MCC	AUC
Baseline (subset with RF)	42.636%	0.335	0.426	0.373	0.121	0.422
Naive Bayes	62.857%	0.622	0.629	0.623	0.447	0.801
LibSVM	57.143%	0.700	0.571	0.532	0.380	0.633
MLP	66.038%	0.647	0.660	0.648	0.502	0.792
SMO	68.571%	0.671	0.686	0.668	0.528	0.795
J48	60.377%	0.717	0.604	0.697	0.422	0.684
J48graft	62.264%	0.589	0.623	0.627	0.500	0.729
Optimized forest	64.151%	0.564	0.642	0.591	0.420	0.835
RF	65.714%	0.605	0.657	0.615	0.457	0.845
MOEFC	74.286%	0.774	0.743	0.767	0.689	0.789
AdaBoostM1(NB)	69.231%	0.746	0.692	0.701	0.564	0.768
Bagging	65.714%	0.605	0.657	0.629	0.472	0.777
Voting	73.585%	0.800	0.736	0.696	0.624	0.862

**Table 7:** Predictive performance of the selected model in course A Extended.

	ACC	Precision	Recall	F-Measure	MCC	AUC
Baseline (subset with RF)	76.974%	0.832	0.770	0.761	0.699	0.931
Naive Bayes	73.494%	0.725	0.735	0.709	0.509	0.906
LibSVM	81.928%	0.820	0.819	0.819	0.672	0.835
MLP	82.530%	0.829	0.825	0.827	0.689	0.904
SMO	81.928%	0.816	0.819	0.816	0.665	0.835
J48	82.530%	0.826	0.825	0.825	0.680	0.860
J48graft	81.373%	0.815	0.814	0.814	0.742	0.904
Optimized forest	81.928%	0.821	0.819	0.819	0.669	0.915
RF	83.133%	0.830	0.831	0.831	0.689	0.929
MOEFC	75.301%	0.786	0.753	0.693	0.575	0.736
AdaBoostM1	82.530%	0.827	0.825	0.823	0.692	0.880
Bagging	81.325%	0.814	0.813	0.811	0.654	0.904
Voting	83.735%	0.836	0.837	0.833	0.699	0.896

**Table 8:** Predictive performance of the selected model in course B Extended.

	ACC	Precision	Recall	F-measure	MCC	AUC
Baseline (subset with RF)	79.518%	0.796	0.795	0.794	0.622	0.905
Naive Bayes	77.362%	0.783	0.774	0.772	0.685	0.916
LibSVM	84.449%	0.853	0.844	0.846	0.779	0.885
MLP	89.216%	0.897	0.892	0.893	0.849	0.974
SMO	88.235%	0.888	0.882	0.884	0.839	0.930
J48	85.294%	0.856	0.853	0.854	0.801	0.927
J48graft	85.294%	0.854	0.853	0.853	0.798	0.918
Optimized forest	88.475%	0.891	0.889	0.890	0.853	0.972
RF	89.216%	0.900	0.892	0.894	0.855	0.983
MOEFC	66.667%	0.755	0.667	0.772	0.686	0.761
AdaBoostM1	92.105%	0.924	0.921	0.922	0.891	0.974
Bagging	86.842%	0.894	0.868	0.870	0.821	0.956
Voting	87.254%	0.878	0.873	0.874	0.824	0.954

For course A, most algorithms can achieve good prediction performance above the Baseline after model adjustment. Among them, LibSVM, SMO, MOEFC, Bagging and Voting achieve the highest accuracy of 78.261%, more than 10% higher than the baseline. This accuracy is obtained when the multi-target genetic algorithm used in MOEFC is adjusted to NSGA II. We choose MOEFC, LibSVM, SMO, J48 and naive Bayes as base classifiers in Voting, but its prediction performance is flat with that of the single model MOEFC, perhaps due to MOEFC. The models with the next best performance is AdaBoostM1 (with acc of 73.913%), MLP, J48graft, RF (with acc of 71.698%) and naive Bayes (with acc of 71.698%).

For course B, the prediction accuracy of most models is slightly higher than the Baseline after the model adjustment and MOEFC, Voting, and AdaBoost M1 perform the best. MOEFC ranks first in accuracy, F-measurre and MCC, with 74.286%, 0.767 and 0.689 respectively, and ranks second with 0.789 on AUC. The selected multi-target genetic algorithm in the MOEFC parameters is NSGA II, generation = 50. The Voting with SMO, J48, and MOEFC as base classifiers achieves the best result of 0.682 on AUC and ranks second on acc and MCC with 73.585% and 0.696%, respectively. The predicted performance of AdaBoost M1 with the NB-based classifier is slightly inferior to that of MOEFC and Voting. Combining the indicators, MOEFC achieves the best predictive performance in both courses. Although we consider MOEFC as the base classifier for Voting, Voting does not perform better than a single MOEFC.

For the course A Extended, which contains more students’ learning data in the middle and late stages of the pandemic, most algorithms can achieve good prediction performance above the Baseline after model adjustment. Among them, Voting achieved the highest accuracy of 83.735%, followed by the RF with 83.133% accuracy, which was about 6% higher than the baseline. The models with the next best performance are MLP, J48 and AdaBoostM1, achieving about 82% accuracy. We selected LibSVM (with polynomial kernel function), SMO, and J48 as the base classifiers in Voting. In addition, we chose naive Bayes as the base classifier of AdaBoostM1, which obviously obtains a significant performance improvement over the single classifier naive Bayes.

For the course B Extended of the same data sizes, most algorithms also achieve good prediction performance above the Baseline after model adjustment. AdaBoostM1 achieves highest accuracy of 92.105%, followed by MLP and RF, which are about 10% higher than the baseline. We choose J48 as the base classifier for AdaBoostM1 and obtained better predictions than the single classifier J48. We also choose LibSVM, SMO, and J48 as the base classifiers in Voting.

#### RQ2: feature importance

Tables 9 and 10 present the results of the experiment on the used feature selection algorithm in both courses.

**Table 9:** Important features in Course A.

Feature slection	Feature	Subset/score
CorrBF/CorrGS	Videojob_time	$\backslash$
	Class_id	
	Job_num	
CORR	Videojob_num	0.370
	Videojob_time	0.370
	Job_num	0.350
	PV	0.300
	Class_id	0.060
InfoGain	Class_id	0.567
	Major_id	0.359
	Job_num	0.208
	Videojob_num	0.196
	Videojob_time	0.196
GainRat	Job_num	0.386
	Videojob_num	0.282
	Videojob_time	0.282
	PV	0.185
	Class_id	0.094
ReF	Major_id	0.224
	Department_id	0.219
	Clazz_id	0.171
	Class_id	0.097
	Videojob_num	0.064

**Table 10:** Important features in Course B.

Feature slection	Feature	Subset/score
CorrBF/CorrGS	Exam_avgscore	$\backslash$
	Class_id	
CORR	Test_avgscore	0.263
	Exam_avgscore	0.252
	Test_num	0.244
	Job_num	0.193
	Special_time	0.191
InfoGain	Class_id	0.918
	Major_id	0.639
	Exam_avgscore	0.260
	Department_id	0.173
	Test_avgscore	0.168
GainRat	Exam_avgscore	0.313
	Test_avgscore	0.171
	Class_id	0.154
	Major_id	0.124
	Department_id	0.047
ReF	Department_id	0.092
	Clazz_id	0.074
	Major_id	0.036
	Test_avgscore	0.030
	Exam_avgscore	0.029

For course A, based on the feature selection algorithms above, videojob_time, videojob_num, job_num, class_id and clazz_id have a great influence on students’ performance. For course B, exam_avgscore and test_avgscore are more important, followed by campus-related attributes. Combining the two courses, students’ completion of learning tasks, stage tests and their classes can influence their academic performance. Actually, the feature categories we use differ from those used in other studies. In reviews conducted inrelated work (Shafiq et al., 2022; Roslan & Chen, 2022), the large proportion of the features are demographic data, socio-economic data, family-related data, pre-university data, followed by historical performance, attendance, LMS Log data, and so on. It also suggests that these key features can help teachers to efficiently monitor students’ learning status during the teaching process and then give guidance duly.

#### RQ3: interviews for practical significance

The results of the students’ and teachers’ ratings of the features in the two courses are shown in Tables 11 and 12, respectively. Additionly, the teachers interviewed, 22 in total, express any and additional explanations they have about the importance of these features through additional dialogue in the process of selecting the important features.

**Table 11:** Results of interviews on important features in Course A.

	1st	2nd	3rd	4th	5th
Student	Videojob_num	Videojob_time	Department_id	Job_num	Class_id
228 (person)	207	184	173	169	154
Teacher	Videojob_time	Job_num	Clazz_id	Videojob_num	Major_id
22 (person)	20	19	17	15	13

**Table 12:** Results of interviews on important features in Course B.

	1st	2nd	3rd	4th	5th
Student	Exam_avgscore	Test_avgscore	Department_id	Class_id	Job_num
129 (person)	118	96	91	73	65
Teacher	Test_avgscore	Exam_avgscore	Clazz_id	Major_id	Videojob_num
22 (person)	21	20	16	13	12

Obviously, the results of students’ and teachers’ choices of important features in the interviews are similar to those of RQ2. In Course A, 91.5%, 80.7% and 74.1% of students think that the number of videos watched (videojob_num), the time spent watching videos (videojob_time), and the number of task points completed (job_num) can affect the final learning performance, respectively. In the case of intense online learning, most of the content of the course is taught in the form of video lectures and document viewing. The importance of these features is also recognized among the teacher community. Teachers consider the amount of time spent watching the video more important than the amount, but only if the students are not distracted during this time period. On the other hand, about 75.9% and 67.5% of students agree on the importance of their college (department_id) and administrative class (clazz_id) in terms of campus attribute categories. This is because different colleges distinguish the mindset of the student body and the level of student understanding varies between administrative classes. Similarly, 77.3% and 59.1% of the teachers agreed more on the importance of teaching classes (clazz_id) and majors (major_id). This is because teachers are able to identify significant differences in classroom climate between teaching classes, and these may influence what students gain in each class. As with colleges, majors, on the other hand, triage students more specifically.

Among the group of students in Course B, 91.5% and 74.4% of the students believe that the results of the stage test (exam_avgscore) and chapter test (test_avgscore) contribute to the final performance, followed by the number of task points completed (50.4%). This is because students include certain knowledge points in their usual stage and chapter tests from the perspective of final review. These two features are also recognized as important by 90.9% and 95.5% of the teacher group, respectively. In addition, the number of videos watched (videojob_num) is considered more important from the teachers’ perspective than the number of ordinary task points completed (job_num). This is because teachers believe that in the process of learning knowledge points, videos contain more detailed explanations about the knowledge points and content than watching static texts, and students are more receptive to this. On the other hand, the findings of the features in Campus Attributes in Course B are almost the same as in A.

In summary, the results of the student and teacher interviews are mostly consistent and similar to the findings obtained in RQ2.

## Discussion

### Model analysis for RQ1

It is worth noting that with the larger data volume (Tables 7 and 8), only MOEFC does not perform well, compared with the integrated classifiers AdaBoostM1, Voting achieved good prediction performance, which is very different from the results in Tables 5 and 6. That is, MOEFC is more suitable for small-scale datasets, and integrated classifiers are more suitable for large-scale such datasets. MOEFC constructs a fuzzy rule based classifier by using the ENORA or NSGA-II, both of which elitist Pareto-based multi-objective evolutionary algorithm, configured to maximize area under ROC curve or minimize the number of fuzzy rules of the classifier. Unlike the single objective optimization technique, NSGA-II simultaneously optimizes each objective without being dominated by any other solution. As a decision tree, J48 has good generalization ability and are robust to noise, providing high performance for relatively small computational effort by using divide and conquer approach. This indicates that few complex interactions are present among features we used, otherwise it will not perform well. Also, without proper pruning decision tree can easily over fit, which is the problem the random forest can handle. RF still can maintains accuracy when the significant dimension of data considered absent. MLP is robust to irrelevant input and noise. Generally, the size of the hidden layer should be determined carefully because of the huge impact on the prediction. An underestimation will lead to poor approximation whereas overestimation will lead to over-fitting and generalization error. Therefore, we choose the average of the feature number and classes hidden layers. AdaBoost, which achieves relatively good prediction results in all situations, is a large margin classifier implicitly optimizing sample margins, and adaptable to training error of each base classifier. Even after the number of iterations increases to a degree that training error reaches zero, AdaBoost still maximizes margin of training samples as much as possible. Voting takes the advantages of each classifier and make up for the disadvantages to give the most suitable prediction results.

**Finding 1.** We prove the validation of the built models and the maintenance of same score even with higher dataset. In the selected models, MOEFC is more suitable for prediction on small-scale datasets, while AdaBoostM1 and Voting are more suitable for larger datasets.

### Feature categories analysis for RQ2

Since the features we used and the results of the important features are different from other studies, we have made comparisons with other datasets. These datasets have different categories of features, and we classify the features in these datasets according to our rules, including demographic information, learning behavior, and campus attributes. The features that could not be classified are classified as “Other”. Considering the different experimental settings of the study, we include some other student achievements that have an indirect effect on the final prediction target in the “Learning Behavior” category. The features of the dataset, after reclassification, are shown in Table 4.

It can be found that learning behaviors and campus attributes account for a lower percentage than demographic and personal information. However, these features are often instructive and can be improved in the teaching and learning process (Adnan et al., 2021; Waheed et al., 2020; Rizvi, Rienties & Khoja, 2019; Song & Li, 2021; Tomasevic, Gvozdenovic & Vranes, 2020). For example, OULAD, one of the widely used publicly available datasets, has been the subject of many other studies. Similar conclusions have been also drawn in nearly every one of them that the models obtain good predictive performance and that these features are important influences on student learning performance. However, OULAD involves a large number of demographic data, and only Date and Sum_click are directly reflective of student behavior, excluding two ratings in the “Learning behavior” category. In the case of high-intensity online learning, however, it is the details of students’ learning that perhaps can provide more guidance and advice for teachers, and the features including these details we call them dynamic features, namely campus attributes and (online) learning behavior. In contrast, those may can not be adjusted according to the situation of students in the later stage we call static features, such as student information and demographic information. In addition, Tomasevic, Gvozdenovic & Vranes (2020) identified the best combination of input data types as Engagement and Performance through classification and regression experiments on this dataset. In other words, the use of demographic data has no significant effect on the accuracy of the predictions.

The features we chose pay more attention to those dynamic features and those related to the learning environment. The advantage of this is that compared with static features, dynamic features can enable students to find learning behaviors that may have greater influence on grades in the learning process and further improve them. At the same time, teachers can adjust the pace and content of classes according to the campus features of these classes, observe the students’ specific learning behavior and task completion, and give personalized guidance. This is because these behavioral features and learning atmosphere which are related to students’ personal learning situation can be dynamically adjusted in the teaching process, and students’ learning efficiency can be improved.

**Finding 2.** For the same course, the results of different feature selection algorithms are almost the same. Without considering static features such as demographic data, in general, students’ learning behavior and stage test scores have an important influence on students’ learning performance. Furthermore, the student campus attributes have a partial impact on the final outcome in both courses, which indicates that the learning atmosphere is also important to the students’ learning process.

### Practice analysis for RQ3

As two actors in the classroom, students and teachers, provide feedback and added different perspectives on the importance of these characteristics. In addition, these teachers interviewed also indicate in additional additions to the selected characteristics that teachers need to be updated as soon as possible on the instructional guidance that students need for online learning, due to the fact that online learning is a new mode of instruction compared to traditional classroom instruction, especially in the context of the COVID-19 pandemic. In other words, it is not clear to teachers whether and how much of the instructional program from the original instructional model is applicable to the new instructional model. For teachers during the pandemic of highly intense online learning, relevant campus attributes and dynamic student learning behaviors are often of greater concern, which may be beneficial to guide students and improving the quality of online instruction. We will then explain these important features in detail.

1. **The completion status and quality of the students’ online tasks can directly reflect their mastery of the course content and knowledge.** The number of videos watched, video viewing time and the number of task points completed in students’ online learning behavior reflect the number and frequency of students’ browsing course contents from the side. This is because most of the knowledge points are covered in video task points and general task points. Teachers can adjust the course progress and urge students to finish it as soon as possible based on the number of videos watched and the completion of task points. Longer video viewing time means more learning and thinking process for students. However, since the level of understanding of the content explained and the time required to absorb the knowledge points varies from person to person, it is not possible to generalize. In addition, there are students who watch videos for a long time but are not in front of the screen.
2. **The learning environment and atmosphere in which students are located on university can indirectly reflect the appropriate teaching style for different students.** In the absence of offline face-to-face classroom and campus activities, these characteristics of the students’ classes and teaching classes can enable teachers to provide different and appropriate teaching styles for the class based on the learning atmosphere of the teaching class. For example, for highly active classes that are more interactive, teachers can provide more opportunities for students to discuss the class content in greater depth. For low activity classes, teachers can increase the frequency of class communication and add class sessions such as teacher-student Q&A and group discussions.
3. **The stage assessment results can reflect the degree of students’ mastery and integration of what they have learned by means of knowledge output.** The performance and completion of the stage tests can indirectly reflect the students’ learning of some knowledge contents in a certain period of time. Based on this, teachers can identify students’ learning in time and also adjust the difficulty of the test and regulate the pace of the course. At this time, teachers can keep track of the students’ learning progress, and know the students’ mastery of knowledge. In addition, the importance of history grades on students’ learning performance has been pointed out in some studies, but the description of relevant factors such as stage tests is not mentioned.

On the other hand, these important features can also correspond to the physical education. In terms of the task completion rate, teachers in online teaching check students’ completion rate and quality, instead of collecting students’ homework regularly to understand the degree of students’ knowledge assessment. For stage ability assessment, the difference between online teaching and offline teaching actually lies in the different media used (paper/electronic), but sometimes online teaching is more inclusive of the form in which students submit their works. For the teaching atmosphere, online teaching can further provide space for students to freely combine and play their abilities, so as to better teach students in accordance with their aptitude.

**Finding 3.** We have further validated the RQ2 findings, and then provide explanatory notes from the three aspects of students’ online learning behavior, learning atmosphere and stage assessment results. Teachers can also provide suggestions based on these features in class instruction to point students in clear directions for improvement in the learning process. These are things that previous static features could not do in practice due to their unimprovable nature.

## Threats to validity

### Construct validity

Threats to external validity lie in the reasonability of the used performance metrics. In this work, four metrics are applied to comprehensively evaluate the performance for students’ final exam grade prediction, including ACC, precision, MCC, recall, F-measure, MCC and AUC. These can make the analysis of our experimental results more rigorous.

### Internal validity

The major threat to internal validity is the implementation mistakes. In our experiments, to relieve this, we take full use of the off-the-shelf implementation provided by WEKA to implement the studied six feature selection techniques and 12 classification models to avoid the potential mistakes.

### External validity

The major threat is that the universality of our study results. In this work, our dataset is selected from two similar courses that are in the COVID-19 pandemic. Due to the variability between courses, this cannot completely replace most of the course data. We will collect more data of different types of courses on student online learning and experiment with it to enhance the generalization of our work.

In addition, the model that performs well on this dataset may not highlight its advantages on other courses datasets, because different datasets contain different structures and use different features. For this, the classification models we use come from six families and the applied feature selection techniques come from two families, which enables the diversity and representativeness of the research objects. This helps to improve the universality of the results. Thus, these models perform well on the dataset containing students’ learning behavior and campus attributes, which can provide help for teachers and students in the teaching process, and the conclusion has proven to be useful and reliable in practice.

On the other hand, the class imbalance issue of the data which we do not consider may affect our experimental results. An extra study on the effects of different class imbalance methods on the performance of student learning performance prediction models will be conducted.

## Conclusion

This article predicted student final learning performance through dynamic feature-driven datasets related to student learning behavior and campus attributes. After verifying the models on the basis of our student’s learning datasets, we further identified important features and factors affecting learning performance. The results showed that most of the selected models could give good classification results and outperforms the baseline. MOEFC is more suitable for prediction on small-scale datasets, while AdaBoostM1 and Voting are more suitable for larger datasets. Furthermore, we reasoned that the features used could reflect that some student learning behavior and campus attributes had important effects on student learning performance, such as completion of different task points, stage test scores, and the student’s class. Compared with the features in other literature, campus attributes such as stage tests and students’ classes can also be associated with learning performance in this condition, which is somewhat similar to the conclusions drawn from physical offline education. In conclusion, we can provide students and teachers with learning guidance and improve the quality of classroom teaching through these dynamic and adjustable features. Additionally, we suggest that teachers can monitor students’ progress and review tasks based on the completion of their online learning assignments, adjust the classroom atmosphere to suit the different classes, and intervene earlier in the learning process to improve students’ mastery of the content based on their stage test results.

Future work includes: (1) collecting more various course data to build a larger and more comprehensive students’ learning dataset, (2) improving the prediction performance and the universality of the models on the other datasets consisting of multiple types of courses, (3) using other approaches accounting for the influence of dynamic features on learning performance.

## Supplemental Information

10.7717/peerj-cs.1699/supp-1 Supplemental Information 1 The students’ learning data in course A. Students’learning behavior, campus attributes and final exam score.

10.7717/peerj-cs.1699/supp-2 Supplemental Information 2 The students’ learning data in course B. Students’ learning behavior, campus attributes and final exam score.

10.7717/peerj-cs.1699/supp-3 Supplemental Information 3 The students’ learning data in course A Extended. Students’ learning behavior, campus attributes and final exam score.

10.7717/peerj-cs.1699/supp-4 Supplemental Information 4 The students’ learning data in course B Extended. Students’ learning behavior, campus attributes and final exam score.

10.7717/peerj-cs.1699/supp-5 Supplemental Information 5 The experimental engineering code. How to prepare the weka environment and the description of two experiments of Prediction and Feature Selection.

## Additional Information and Declarations

### Data Availability

The following information was supplied regarding data availability: The raw data and weka engineering files and description are available in the Supplemental Files.
